# Proposing a new solution for marine debris by utilizing on-board low-temperature eco-friendly pulverization system

**DOI:** 10.1038/s41598-021-03757-z

**Published:** 2021-12-21

**Authors:** Dong-Ha Lee, Sungkyun Park, Hee-Tae Kim, Jeong-Dae Kim, Jeong-Hyeon Kim, Seul-Kee Kim, Jung-Kwan Seo, Pung-Keun Song, Jeong-Eun Oh, BuHyun Youn, Gyung-Min Choi, Dong-Ha Lim, Jae-Myung Lee

**Affiliations:** 1 Department of Naval Architecture and Ocean Engineering, Pusan National University, Busan, 46241 Korea; 2 Department of Physics, Pusan National University, Busan, 46241 Korea; 3 Hydrogen Ship Technology Center, Pusan National University, Busan, 46241 Korea; 4 Department of Materials Science and Engineering, Pusan National University, Busan, 46241 Korea; 5 Department of Civil and Environmental Engineering, Pusan National University, Busan, 46241 Korea; 6 Department of Biological Sciences, Pusan National University, Busan, 46241 Korea; 7 Department of Mechanical Engineering, Pusan National University, Busan, 46241 Korea; 8 Korea Institute of Industrial Technology, Busan, 46938 Korea

**Keywords:** Ocean sciences, Engineering

## Abstract

Developing an effective and efficient recycling process for marine debris (MD) is one of the most urgent issues to maintain environmental sustainability on Earth. However, restricted storage capacities and secondary pollution (e.g., microbial adhesion, putrefaction) limit the proper MD recycling. Here, we proposed a complete eco-friendly low-temperature MD pulverizing system that utilizes excessive liquefied natural gas (LNG) cold energy (LCE) in an LNG propulsion ship to improve the efficiency and effectiveness of MD recycling. The prototype design of the low-temperature pulverization (LTP) system showed that consumable refrigerant (liquid nitrogen) up to 2831 kg per hour could be substituted. Furthermore, with a 20% ship output, 1250 kg of MD could be treated with 363 kg of additional refrigerant. In addition, LTP systems utilizing LCE could increase the storage capacity by more than 10 times compared to bulk MD while minimizing the required energy consumption. To determine the feasibility of LTP for MD recycling, four types of plastics obtained from actual MD from a coastal area in Busan, Korea were classified and tested.

## Introduction

Since the 1970s, marine debris (MD) has increased due to rapid industrialization^1,2^. MD can take a severe toll on biological^3–6^, economic^7,8^, and aesthetic (tourism)^9,10^ factors. Plastic production, which surged with industrialization in the 1950s, exceeded the cumulative production of 8.3 billion tons in 2017. According to Geyer et al., 59% of plastics are left unattended without being recycled or incinerated^11^. These plastics flow naturally into the ocean from land^12,13^. According to Eriksen et al., there are about 85 to 150 million tons of marine plastic debris (MPD) divided into 5 trillion pieces in the world’s oceans, causing severe marine ecological issues^14^. Additionally, due to the COVID-19 pandemic, there are concerns that the increasing use of plastics, including personal protective equipment (PPE), is exacerbating marine pollution^15,16^. The lifetimes of MPD are relatively long and unpredictable. Therefore, they accumulate in the ocean for decades without decomposition^17^. Plastics, washed up onto shores, are broken down by weathering and erosion, causing the indiscriminate production of microplastics with a particle size of less than 5 mm^18^. These microplastics are released into the marine ecosystem and lead to coastal pollution and biological toxicity^19^. Not only that, but they also impact human health; according to Cox et al., an adult male ingests, directly or indirectly, more than 60,000 pieces of microplastics per year^20^. In addition, secondary pollution of MPD owing to the marine environment, such as microbial adhesion, putrefaction, ingestion of marine organisms, leads them to be non-recyclable^1,21^. Currently, many non-profit environmental organizations are finding ways to resolve the issue of MPD distributed in the oceans, especially the Pacific Ocean ^22–26^.

In general, cleaning ships, equipped with facilities to dispose of floating and immersed wastes, collect and process MD^27,28^. Recently, eco-friendly cleaning ships with solar and wind generation systems have been developed. Furthermore, they can handle MD on board by plasma gasification facilities to produce fuel for the ship^29^. In addition, the “Ocean Cleanup” system using natural oceanic forces, collects floating MD in the Great Pacific Garbage Patch (GPGP). It gathers up plastic and ghost nets with the support of a U-shaped arm^30^. However, these ships are limited in terms of their operating radius and time because of the limited size of cargo capable of storing MD. Therefore, increasing the size of the MD cargo hold is a critical parameter that should be considered for better MD recycling. However, these ships are limited in terms of their operating radius and time because of the limited size of cargo capable of storing MD. Therefore, increasing the size of the MD cargo hold is a critical parameter that should be considered for better MD recycling.

In the collection stage, MD is bulky and disadvantageous to store/transport due to its low packing density, which prevents the efficient collection of MD by the aforementioned ships^31^. A method of improving the storage efficiency in a limited cargo hold includes a pulverizing and loading method. In addition, according to the Northwest Pacific Action Plan (NOWPAP), current plastic recycling technologies can be classified into three categories: material recycling (or mechanical recycling), chemical recycling (or feedstock recycling), and thermal recycling (or energy recovery)^32^. Notably, all approaches have common issues in pulverizing bulky MD as a preprocessing step to enhance the portability and readiness for other uses^33,34^. Accordingly, MD, previously processed on the ship, might be transported on land and immediately applied to recycling. However, because of the low melting point of plastic [e.g., thermoplastics (TP)], it is difficult to pulverize plastics into smaller particle sizes. As an alternative, a low-temperature pulverization (LTP) process was proposed to improve pulverizing efficiency^35–38^. Furthermore, there was an attempt to construct a cooling system by utilizing the cold heat from a liquid gas storage tank such as liquefied natural gas (LNG)^39–42^.

Meanwhile, LNG in a cryogenic state is used as an eco-friendly fuel in the transportation industry and as an onshore energy resource. Recently tightened ship emissions legislations (e.g., Tier III requirements of the revised MARPOL Annex VI mandate) have increased the demand for LNG propulsion ships. In this regard, many nations are making great efforts to demonstrate LNG-fueled propulsion systems^43^ since LNG can reduce the energy efficiency design index by 20%^44–46^. Furthermore, about 860 kJ/kg of cold heat is wasted when LNG is vaporized and overheated in a dual-fuel (diesel-LNG) engine ship, a typical LNG propulsion ship type^47^. Therefore, it is advantageous to improve and/or develop a system to maximize the usage of excessive LNG cold energy (LCE). In case of LNG carrier (LNGC), the utilization of excessive LCE, such as through power generation or desalination, can be seen in many studies^39,48^. Furthermore, LCE can be applied to cryogenic power generation systems through the organic Rankine cycle (ORC) and Brayton cycle^49,50^. However, the cold heat generated by LNG propulsion ships is rarely used due to the small capacity of the fuel storage tank compared to the cargo hold of the LNGC.

Therefore, there is a need for a plan to utilize the cold energy from newly built LNG propulsion ships. This study proposes a conceptual design that combines the MD disposal system and the residual cold energy utilization of an LNG propulsion ship to build an eco-friendly and cost-effective LTP system. The amount of additional refrigerant used for freezing MD when the LCE system is operational is also quantified and evaluated in the prototypical ship for collecting MD. Further, LTP on MPD samples collected from a coastal shoreline of Busan, Korea was tested to show the feasibility of the proposed LTP system. As a result, this study shows that (1) LTP systems can be used to treat MD by processing MD into finer particles to improve the ship storage capacity and (2) building LCE-based LTP systems in LNG-fueled propulsion ships can provide an alternative route to improve MD recycling and upcycling to ensure Earth’s sustainability.

## System description

**Figure 1:**
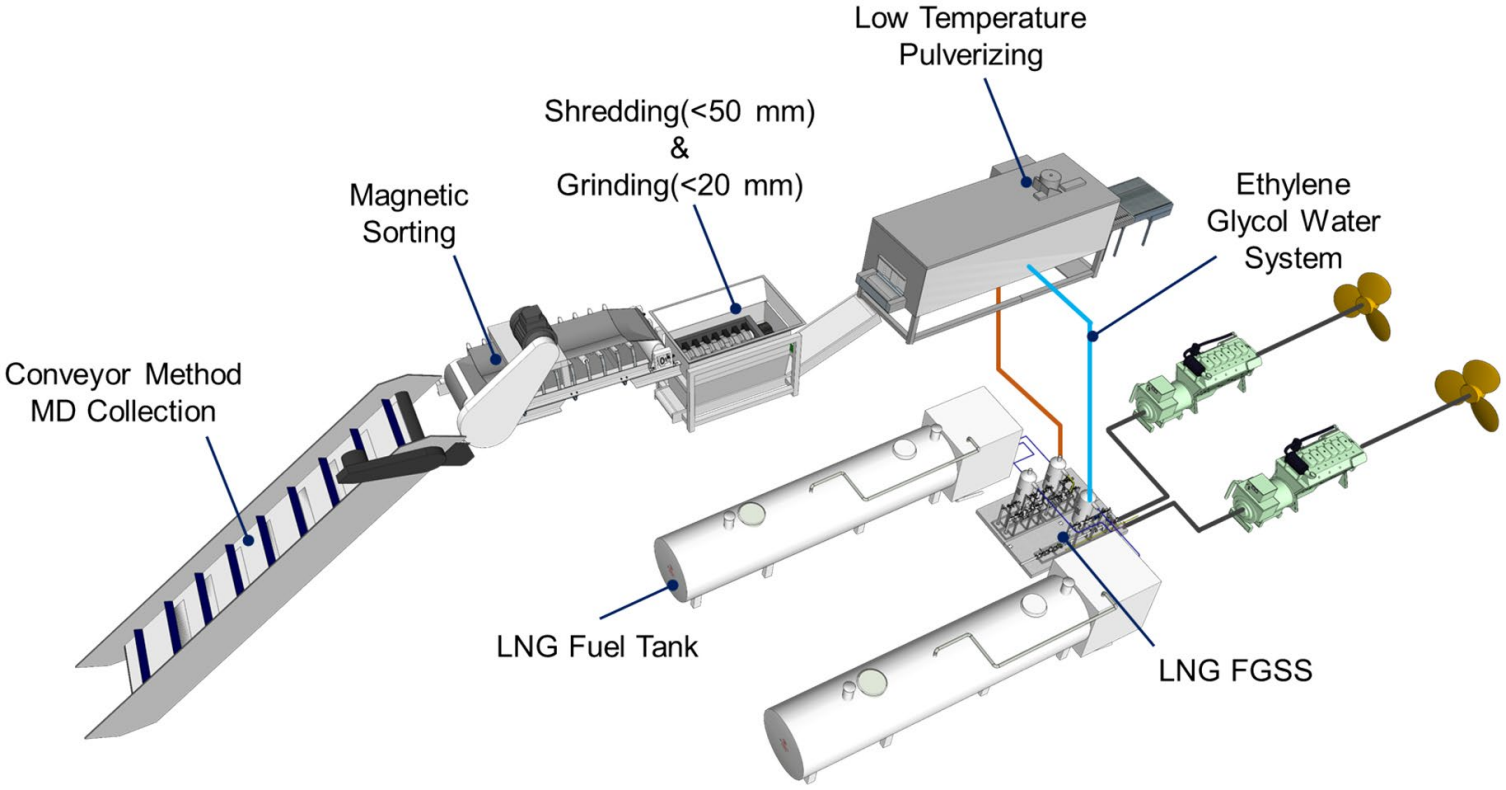
Layout for LNG-fueled propulsion system equipped with an LTP system utilizing LCE.

Pulverization is an essential process for recycling marine waste. It turns processed plastics into different products in a single form, allowing for consistency in subsequent processes. Furthermore, this pre-treatment process can be more economical and efficient if the energy required to collect and preprocess MD is from surplus resources. For example, refrigeration using LCE can reduce initial investment and maintenance costs due to the simplification of facilities. In addition, using the existing refrigerant circulation system for the condensation–expansion process when using surplus LCE does not require additional equipment. Figure 1 shows the layout of the main facilities of an MD collection and cleaning ship equipped with an LTP facility. The facility is divided into two parts. The first is the propulsion part containing the LNG fuel tank. According to the eco-friendly trend in shipbuilding, MD collection and cleaning vessels are using LNG as fuel. LNG in the cryogenic state causes phase changes in the fuel gas supply system (FGSS), resulting in heat exchange. The gas is then combusted to generate the energy needed for power. Propulsion can also be carried out through the direct internal combustion of LNG, but in ships such as ferries, electric propulsion is also applied using an LNG power generator^51,52^. The second is the MD disposal part. In floating MD, collection through a conveyor is effective and can operate at a constant rate to bring the debris from the ocean directly to the storage cargo hold^53^. Furthermore, magnetic separators and dechlorination facilities are included. A detailed description of the pulverization process will be provided later (see Fig. 5).

**Figure 2:**
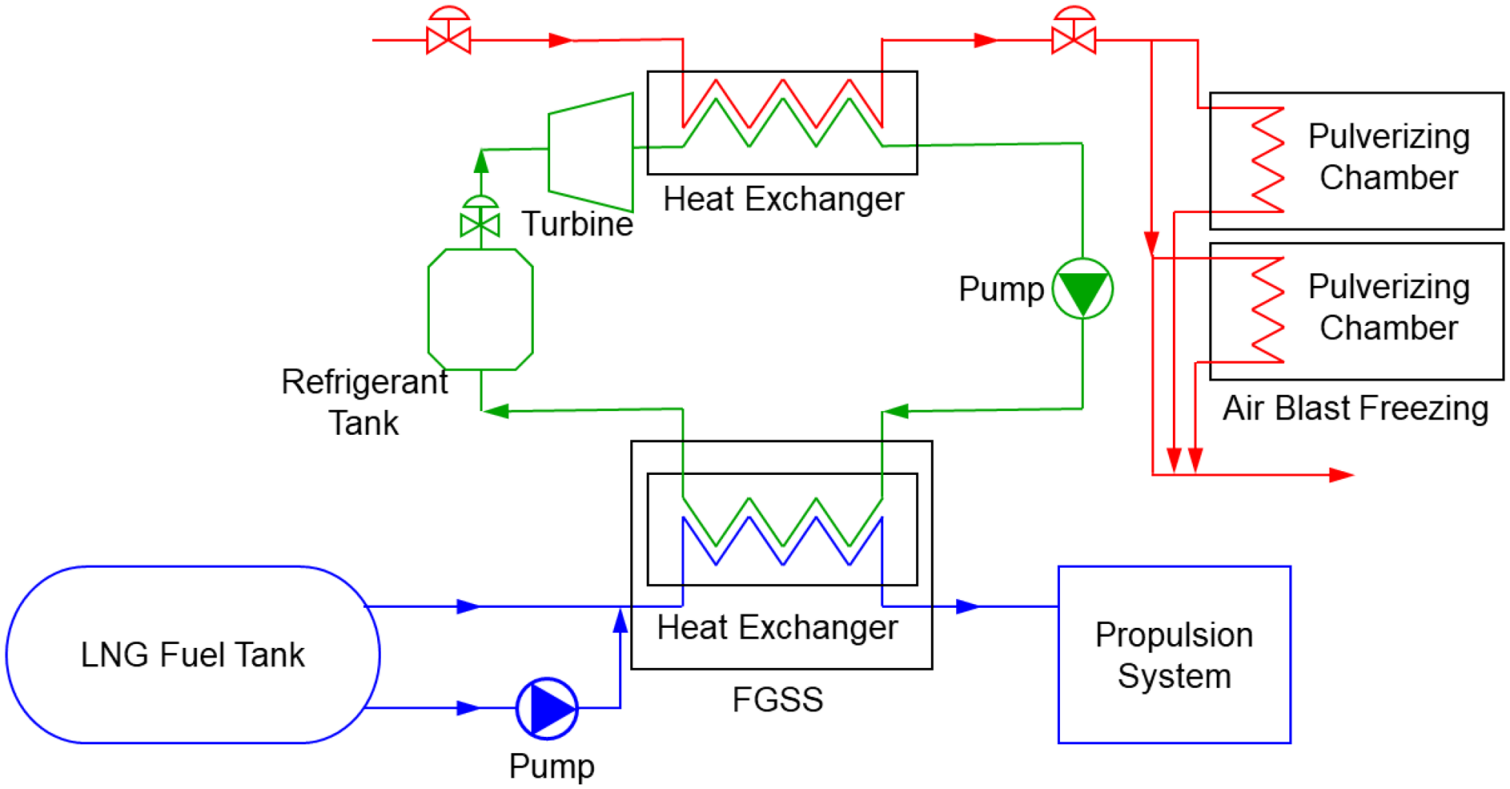
Schematic diagram of proposed LNG-fueled propulsion system with LTP. It contains the LNG propulsion part (blue line), EGW system (green line), and pulverizing chamber with an air-blast freezing system (red line).

Figure 2 shows the details of the proposed schematic diagram of the system for freezing MD for LTP. LNG lowers the temperature of ethylene glycol water (EGW) in the heat exchanger of the FGSS^54^. Ethylene glycol is typically used as a heat transfer medium owing to its low freezing point, which suits the low-temperature condition of the LNG stream^39^. Therefore, cold air with the circulating EGW decreases the temperature of MD via contact (i.e., air-blast method). As a result, MD is frozen to a brittle temperature. Furthermore, this LTP system (upper-right side of Fig. 2) supplies continuous cold energy without a heat exchanger.

To evaluate its potential cooling capacity and feasibility, we constructed a prototypical LNG propulsion cleaning ship with proper parameters. The ship has a cargo capacity of 1300 m^3^ for loading MD and is equipped with an LTP facility capable of handling 20 tons of MD per day. The LTP facility operates in two units for cleaning efficiency, considering an eight-hour workload per day. Table 1 lists the specifications of the prototypical cleaning ship. Based on the ship’s specification, the heat transfer rate for freezing MD is calculated as follows^55,56^:

1 $$\dot{Q}_{MD} = \dot{m}_{LNG} (h_{out} - h_{in} ),$$

where $\dot{Q}_{MD}$ and $\dot{m}_{LNG}$ represent the heat transfer rate in the pulverizer (J/s), and mass flow rate of LNG (kg/s). The *h*_*out*_ and *h*_*in*_ represent specific enthalpy at the outlet and inlet of the heat exchanger (J/kg). In this calculation, the temperature of the LNG at the outlet was fixed at 268 K, and the system assumed adiabatic behavior^57^. Table 2 lists the embrittlement temperature and the specific heat of the test plastics^58–62^. The target temperature for pulverization was assumed to be the ductile–brittle transition temperature (DBTT). DBTT studies on many plastics have been performed. In this study, all plastics were assumed to be polyethylene to calculate the maximum refrigerant needed to reach DBTT. Additionally, to compare the efficiency of cooling systems, refrigerant consumption was calculated for liquid nitrogen (LN_2_). Equation (2) shows the relationship in the amount of refrigerant used for the LTP of plastics^63^;

2 $$MC_{pM} (T_{i} - T_{s} ) = GC_{pR} (T_{gO} - 77.4),$$

where *M* is the flow rate of MD (kg/h m^2^), *C*_*pM*_ is the specific heat of MD (J/kg K), *T*_*i*_ is the inlet temperature of MD (K), *T*_*s*_ is the temperature of MD at the end of the pre-cooling section (K), *G* is the flow of refrigerant (kg/h m^2^), C_pR_ is the specific heat of refrigerant (J/kg K), and T_gO_ is the outlet temperature of refrigerant (K).

**Table 1:** Principal particulars of prototypical cleaning vessel.

Particulars	Specification	Unit
Engine type	Himsen 5H22CDFP	–
Engine rated power	2200	kW
LNG fuel tank	500	m^3^
LNG pressure	5	bar
Design maximum speed	11.5	knots
Cruising distance	2200	NM
Cargo volume	1300	m^3^
Work capacity	20	Ton/day

**Table 2:** Properties of plastics applied to freezing and pulverizing.

Polymer	DBTT (℃)	Specific heat (J/kgK)	References
PA	230	1700	^58^
PE	230	1900	^59^
PET	240	1200	^60^
PP	250	1700	^61^
PVC	270	1250	^62^

If the flow rate of MD is expressed as the ratio of refrigerant flow, the amount of refrigerant needed to pulverize MD (i.e., M/G) can be calculated as follows;

3 $$\frac{M}{G} = C_{pR} (T_{gO} - 77.4)/C_{pM} (T_{i} - T_{s} ).$$

It is worth noting that the available LCE for MD collection and cleaning is limited when the cleaning ship moves at a relatively low speed because of the less excessive LCE. Therefore, it is necessary to determine the MD freezing capacity depending on the speed of a ship, which can be calculated from the fuel consumption. In addition, the fuel capacity of the prototype ship can be determined by calculating the ship speed-dependent fuel consumption (*W*_*LNG*_). Assuming the prototypical ship is equipped with a Himsen engine (Hyundai Heavy Industry, HHI) and its specific gas consumption (SGC) based on maximum continuous rating (MCR) is 163.42 g/kWh, the amount of freezing capacity using LCE per hour (*W*_*LCE*_) according to the output of the ship (*P*_*E*_) is as follows;

4 $${W}_{LCE}=\frac{{h}_{\mathit{out}}-{h}_{in}}{{C}_{pM}({T}_{i}-{T}_{s})}\times SGC\times {P}_{E}.$$

**Figure 3:**
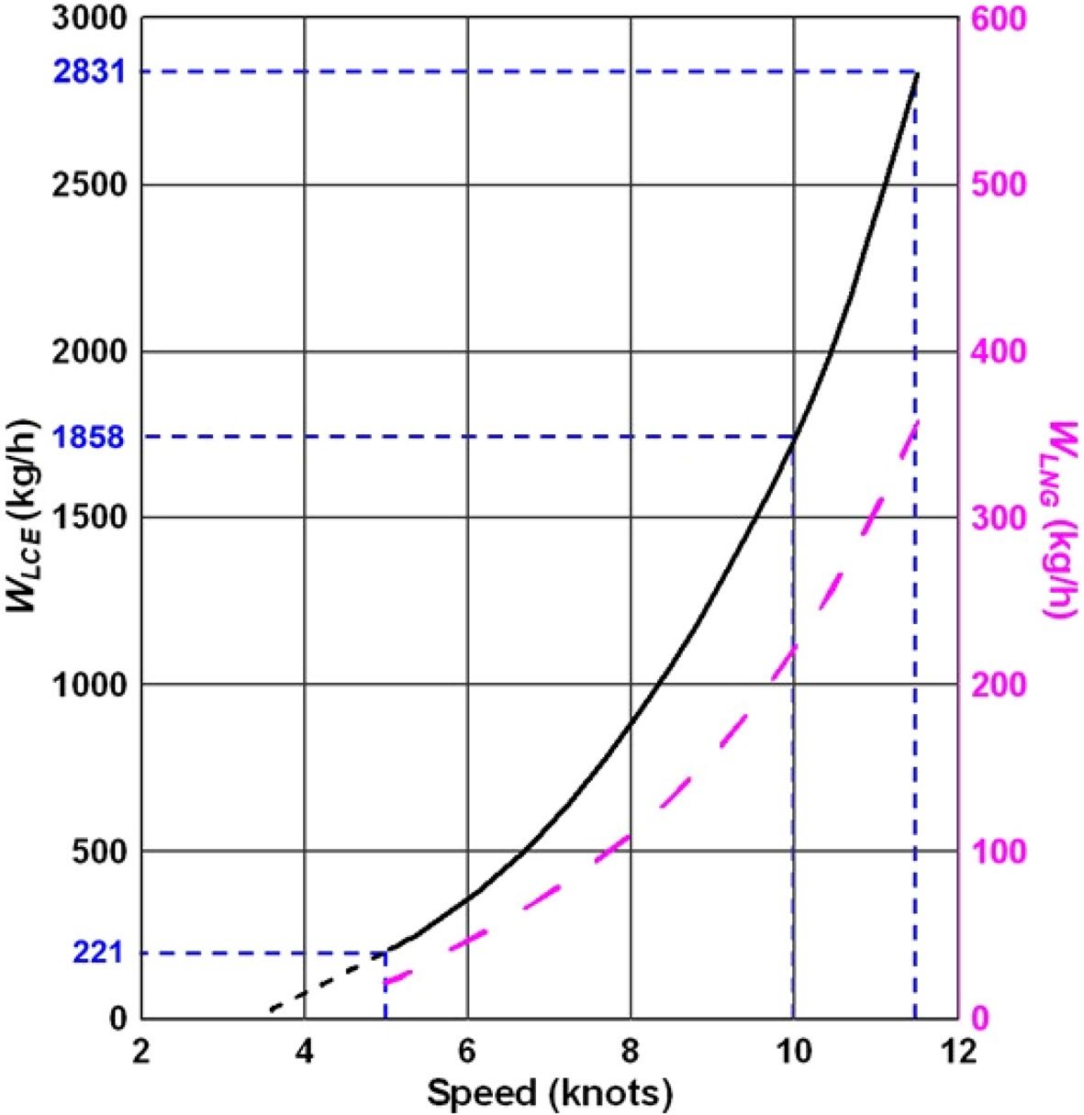
Ship speed dependent maximum MD freezing capacity (solid black curve) and LNG fuel consumption capacity (dotted magenta curve). The horizontal and vertical dotted lines indicate the two distinct points (i.e., low speed and high speed).

Furthermore, *P*_*E*_ is proportional to *v*^*3*^, where *v* is the ship's speed^64^. Figure 3 shows the calculated *W*_*LCE*_ and *W*_*LNG*_ depending on the ship’s speed, *v*. Through the LNG consumption rate (in this case, *W*_*LNG*_), the MD collection range and route can be optimized^65–67^. It is worth noting that the estimated *W*_*LCE*_ based on MCR, which is less than 10% (less than 5 knots in this study), is inaccurate. Therefore, the minimum speed for collecting marine waste is assumed to be 5 knots. In this case, the LTP system can freeze 221 kg of MD per hour using the cold energy of LNG consumed. Furthermore, MD collection and LTP are independent processes, suggesting that two processes can occur simultaneously (i.e., independently) when a ship operates. However, much fuel is consumed when a ship sails at a high output after MD collection. For example, 1858 kg of MD can be frozen per hour at the speed of 10 knots/2831 kg at the design speed. Therefore, more effective LTP can be done at a high speed.

**Figure 4:**
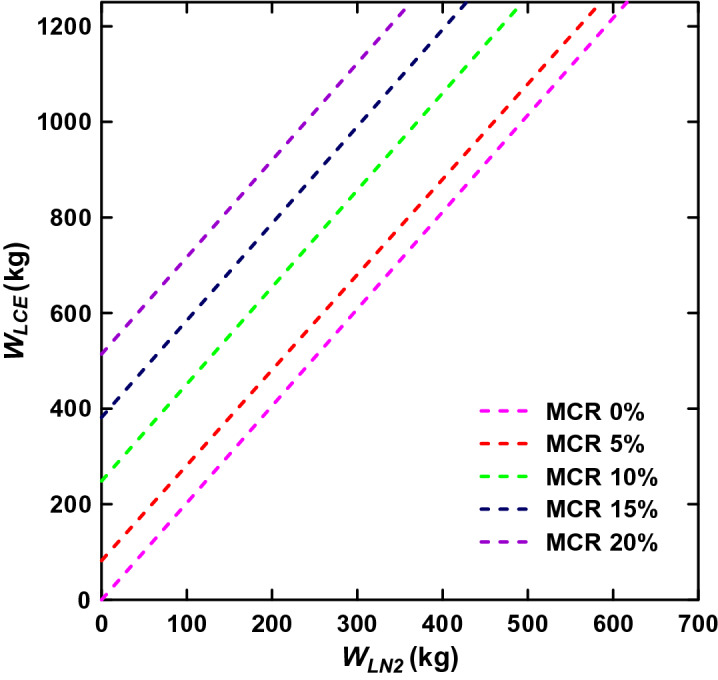
MD freezing capacity compared to liquid nitrogen consumption according to the ship’s output.

In general, MD collection ships need to stay in the ocean for a long time compared to merchant and passenger ships. Therefore, the targeted collection area and LTP throughput should be designed by adjusting the size of the LNG fuel tank. Considering that optimal MD collection is operated at speeds of 5 knots or less, it is possible to freeze up to 250 kg of MD per hour without any additional energy. Therefore, if an additional refrigerant (e.g., LN_2_) is used, the extra MD can be frozen and pulverized. The additional amount of liquid nitrogen (*W*_*LN2*_), needed for overflow MD freezing and pulverizing can be derived as follows from Eqs. (3) and (4):

5 $$W_{LN2} = W_{LCE} \times \frac{M}{G} + MFC,$$

where *MFC* means the minimum freezing capacity according to the MCR. The correlation between *W*_*LN2*_ and *W*_*LCE*_ for various MCR is shown in Fig. 4. The slop (M/G) is constant regardless of the percentage of MCR expected. Suppose the ship is not in operation (i.e., MCR = 0%). Then, MD should be frozen through LN_2_ only. However, if an MD collection ship increases the power output, the LCE replaces LN_2_. For example, it is possible to freeze 246 kg (514 kg) of MD per hour at an output of 10% (20%) MCR without additional refrigerant. The corresponding ship’s speed for each output is 5.34 knots for 10% MCR and 6.73 knots for 20% MCR.

**Figure 5:**
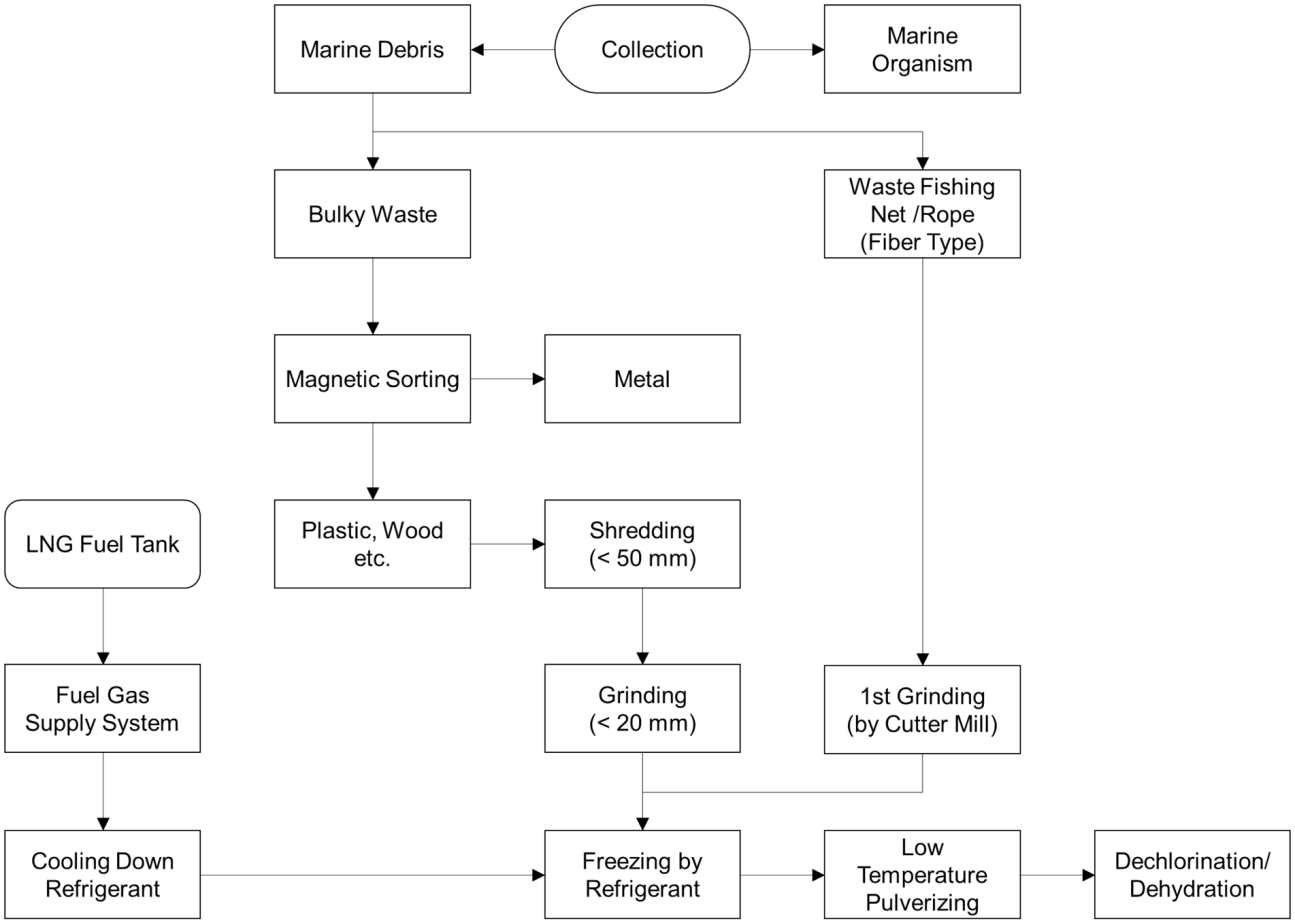
Low-temperature MD pulverizing process utilizing LCE.

Figure 5 shows the detailed LTP process of MD using LCE. The collected waste is classified into MD and marine organisms. Since marine organisms such as echinoderms and seaweeds inhabit the seabed, they should be separated. Further, among the classified MD, fiber-type waste, such as dumped fishing nets or rope, are sorted out because entanglement and overload can be induced in the shredding and grinding process^68^. In addition, floating MD might contain metals and/or other high-density materials^69,70^. In the case of wasted metals, the magnetic separator is used to filter them out. Simultaneously, high-density materials should be separated through a specific gravity sorting process prior to the cutting. The remaining MD is primarily crushed by a shredding machine^71^. The shredding machine has the advantage of a high grinding capacity. However, the ground particle size is relatively large at ~ 50 mm^72^. Therefore, improving the LTP efficiency requires further processing to a particle size of 20 mm or less. To do this, the particles are stored in a low-temperature freezer (e.g., ~ 233 K) for a while prior to the LTP process. To lower the refrigerant temperature in the freezer, the LCE, which is waste energy, is supplied to the FGSS.

**Figure 6:**
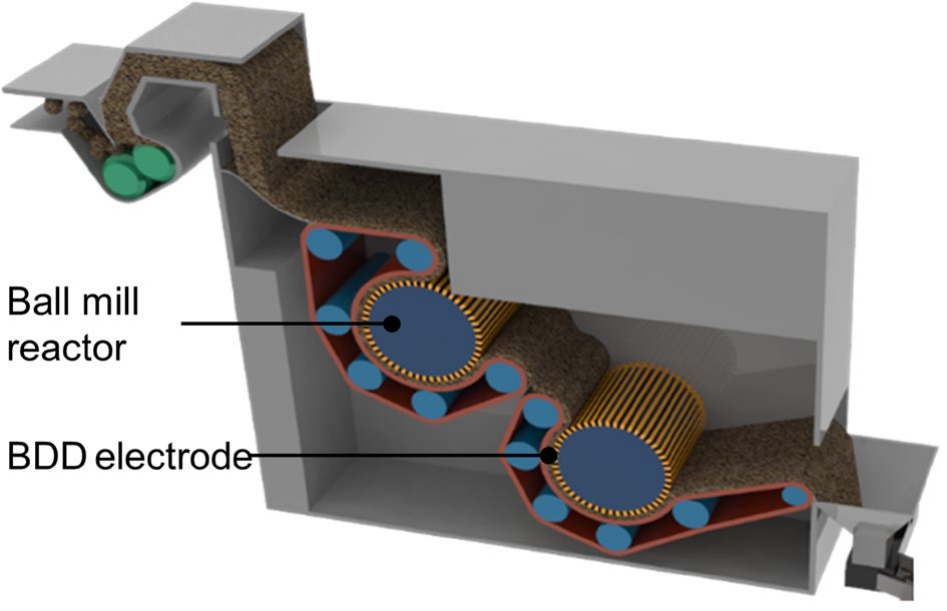
Schematic of the CD equipment. It uses a BDD electrode instead of an IrO_2_ electrode.

Some collected plastic MD contains chlorine. For example, polyvinyl chloride (PVC) is a TP amorphous with a high molecular compound used in various places due to its low price, rigidity, and high immutability^73^. However, since PVC contains chlorine, many toxic substances such as dioxins and furans may be generated during incineration and thermal decomposition. Therefore, a separate dechlorination process is required^74^. In addition, electrochemical treatment is essential due to the high salinity of MD and wastewater generated from the pulverizing process. IrO_2_ electrodes have been widely used for wastewater desalination. However, boron-doped diamond (BDD) electrodes were developed to generate strong oxidizing agents such as OH–. Strong oxidants can react with Cl in plastics (or Cl– of waste seawater) to produce additional oxidants such as hypochlorous acid (HCLO) and perchlorate (CLO_4_^−^), which can remove chlorine. Figure 6 shows the schematics of drum-type capacitive dichlorination (CD) equipment with a ball mill reactor and the detailed chemical process related to dichlorination. Drum-type dechlorination facilities are designed to perform plastic dechlorination treatments at a 470 K or higher temperature with BDD electrodes. The properly crushed MD particles, which have undergone the dechlorination and dehydration processes, can be utilized in two ways: First, they can be used as base materials for construction products and reinforced concrete as part of upcycling efforts. Second, they can be used as fuel resulting from high-temperature gasification. This is a thermal process that converts organic matter into syngas (synthesis gas), primarily made up of hydrogen and carbon monoxide.

## Marine debris pulverization

**Figure 7:**
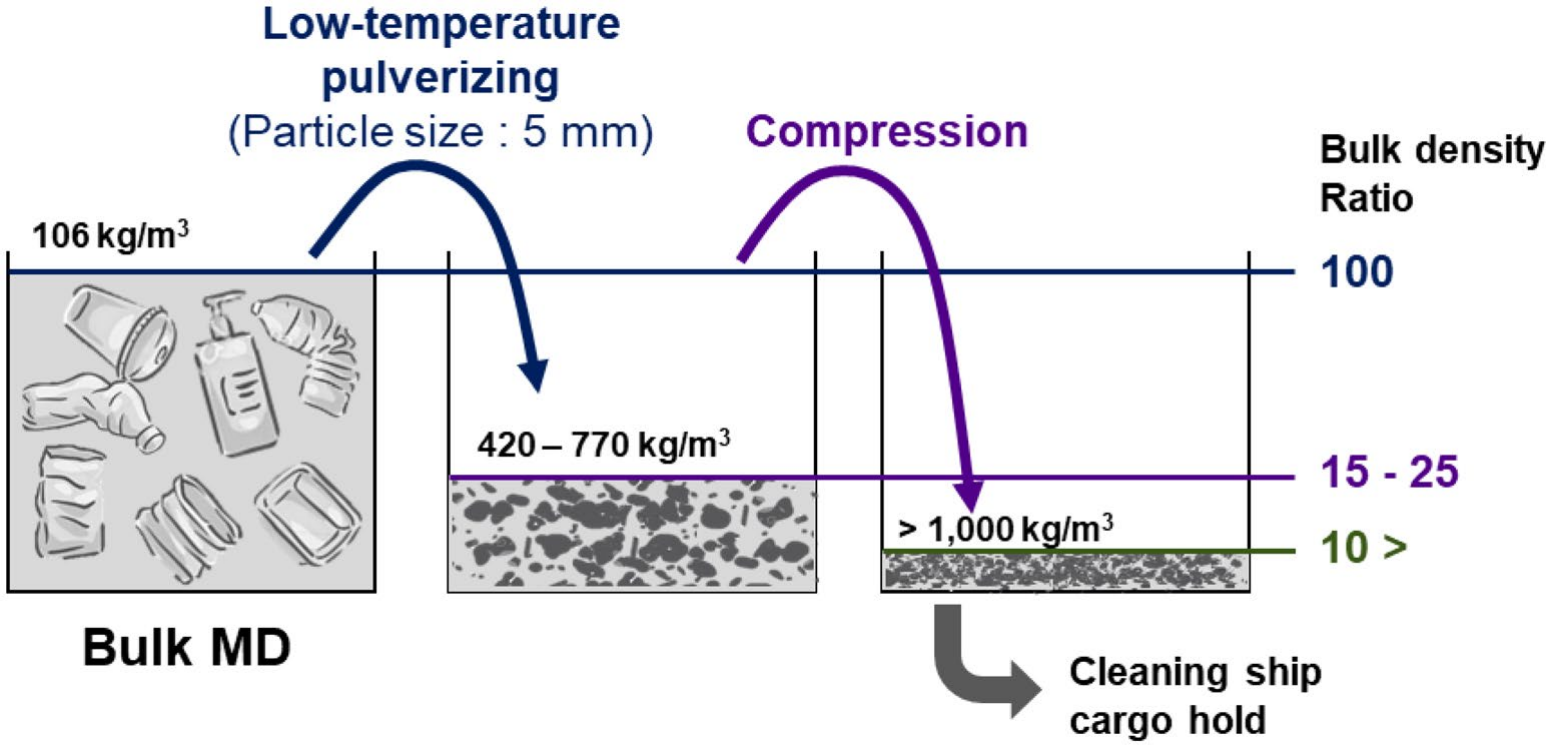
Storage efficiency according to the stages of MD treatment. Bulk MD contains seawater and has poor storage efficiency due to its low bulk density. Even if only the pulverizing process is carried out, it is possible to achieve a storage efficiency of more than five times and is expected to store more than 10 times through compression^69^.

LTP, which is a pre-treatment process for waste recycling, is known to improve storage efficiency. Figure 7 shows the typical bulk MD with a small density (106 kg/m^3^) and a large volume. Therefore, collection bulk MD without processing prevents mass collection. However, pulverizing MD into particles smaller than 5 mm increases the density to 420–770 kg/m^3^, increasing the loading efficiency up to seven times. Furthermore, the additional compression process increases the packing density by more than 10 times. Therefore, an energy-efficient pulverizing (e.g., LTP process) and compression process is essential to enhance a ship’s cleaning capacity and long-term operation.

**Figure 8:**
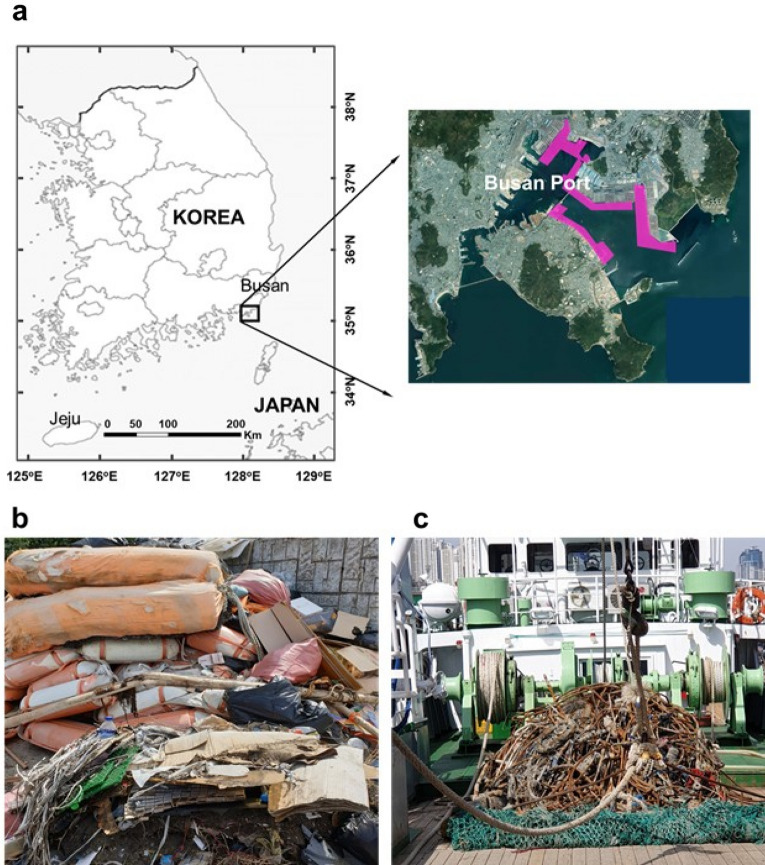
(**a**) Map of South Korea (left) and a satellite image (right) of the area near the Busan port in Busan, South Korea, where the MPD was collected. The map and satellite image is obtained from Matlab R2021b and the free map service provided by the Korea National Geographic Information Institute (https://www.ngii.go.kr/eng/main.do as of November 5th, 2021) (**b**) Photograph of the piled MPD left in a storage house without going through and separating the collected waste. The MPD was collected near the Busan Port as of February 12th, 2020. It comprises household plastics, fishing gear, and buoys. (**c**) The seabed waste, collected near the coast. It is mainly in the form of fibers such as fishing nets or rope.

A practical test to determine the feasibility of the LTP process of TP–MPD was performed. The MPD used for the pulverization test was collected within the range of 4 km off the coast of Busan, Korea as shown in Fig. 8a. A cleaning ship operated by the Korean government collected floating MD (Fig. 8b) and seabed MD (Fig. 8c). As mentioned in “System description”, MD collected by cleaning ships is currently stored in warehouses prior to moving to a landfill or incineration since recycling is inefficient due to contamination and chemical decomposition^75^. In particular, fishing nets and rope from fishing boats, as shown in Fig. 8c, are highly corroded and decomposed, so the recycling cost is very high. Furthermore, the processing procedure is very complicated.

**Figure 9:**
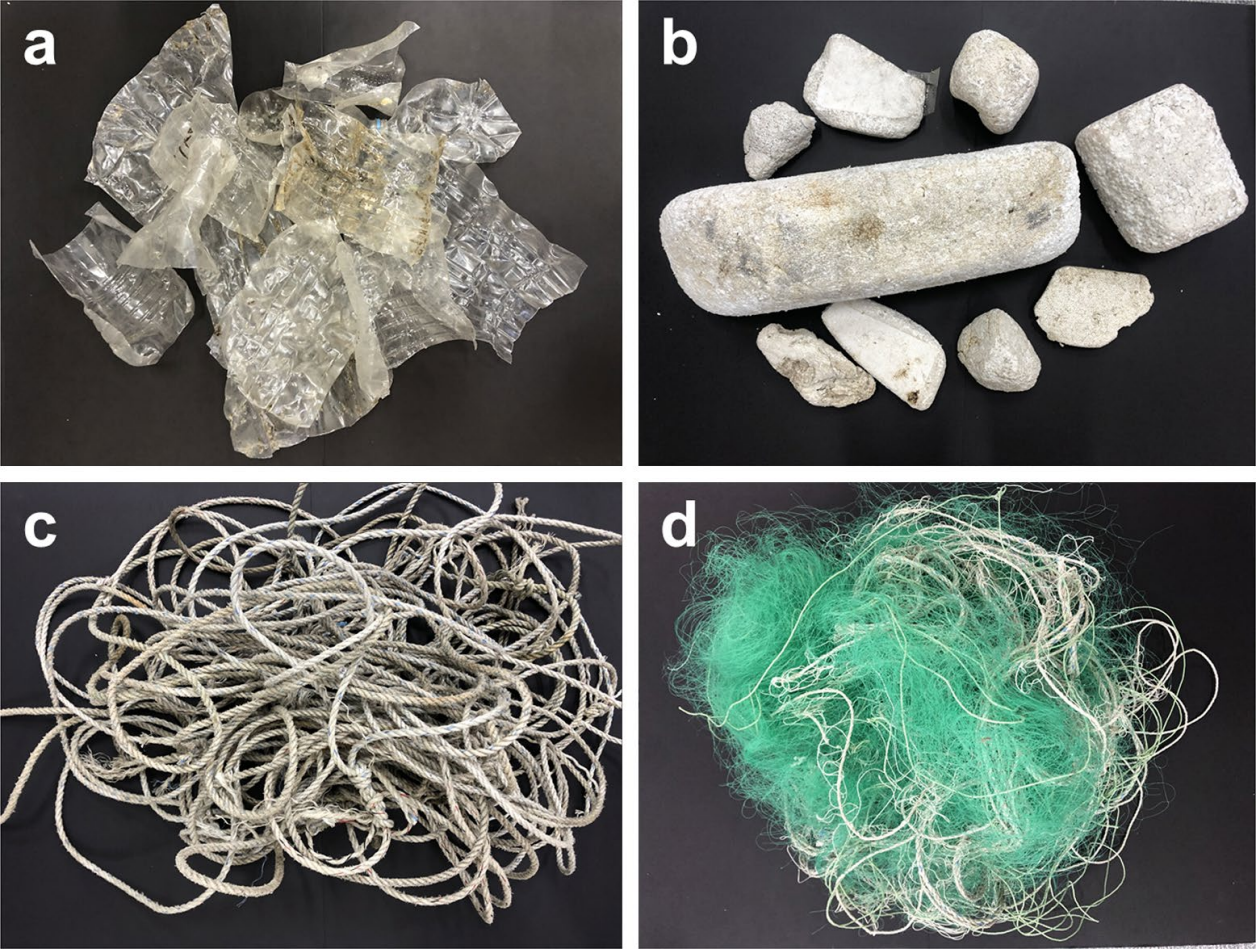
Photograph of classified wastes such as (**a**) plastic bottles, (**b**) buoys, (**c**) rope, and (**d**) fishing nets for LTP tests.

Randomly collected floating MD from a conveyor method was primarily classified by manual labor into four materials: polyethylene terephthalate (PET), expanded polystyrene (EPS), polyamide (PA), and polypropylene (PP) (Fig. 9a–d). PET was acquired through land-based household waste, and EPS was chosen from buoys among the floating waste. PA and PP were obtained from abandoned nets and rope among those dumped from fishing boats. Furthermore, classified MD was confirmed through Fourier-transform infrared spectroscopy (FT-IR) analysis that allowed a comparison with reference materials. In general, ultrasonic mill, jet mill, and ball mill are used to make fine particles^76–79^. However, it is advantageous to select a cutter mill or an impactor mill for large pulverizing volumes such as waste. Therefore, the impactor mill, which had two opposite blades at 24,000 RPM, was used to pulverize the material while circulating LN_2_ to maintain a low temperature. The temperature was set to 220 K followed by an hour of pre-cooling before pulverization. In addition, the refrigerant was continuously supplied during pulverizing to prevent internal temperature rise during the use of the high-speed rotating pulverizer. The temperature of the inside of pulverizer was monitored in real-time to avoid plastic melting. Since the temperature of pulverizing MD could not be measured, the inlet and outlet of pulverizer were monitored, respectively, and the internal environment was maintained at 220 K by adjusting the flow rate of the refrigerant.

**Figure 10:**
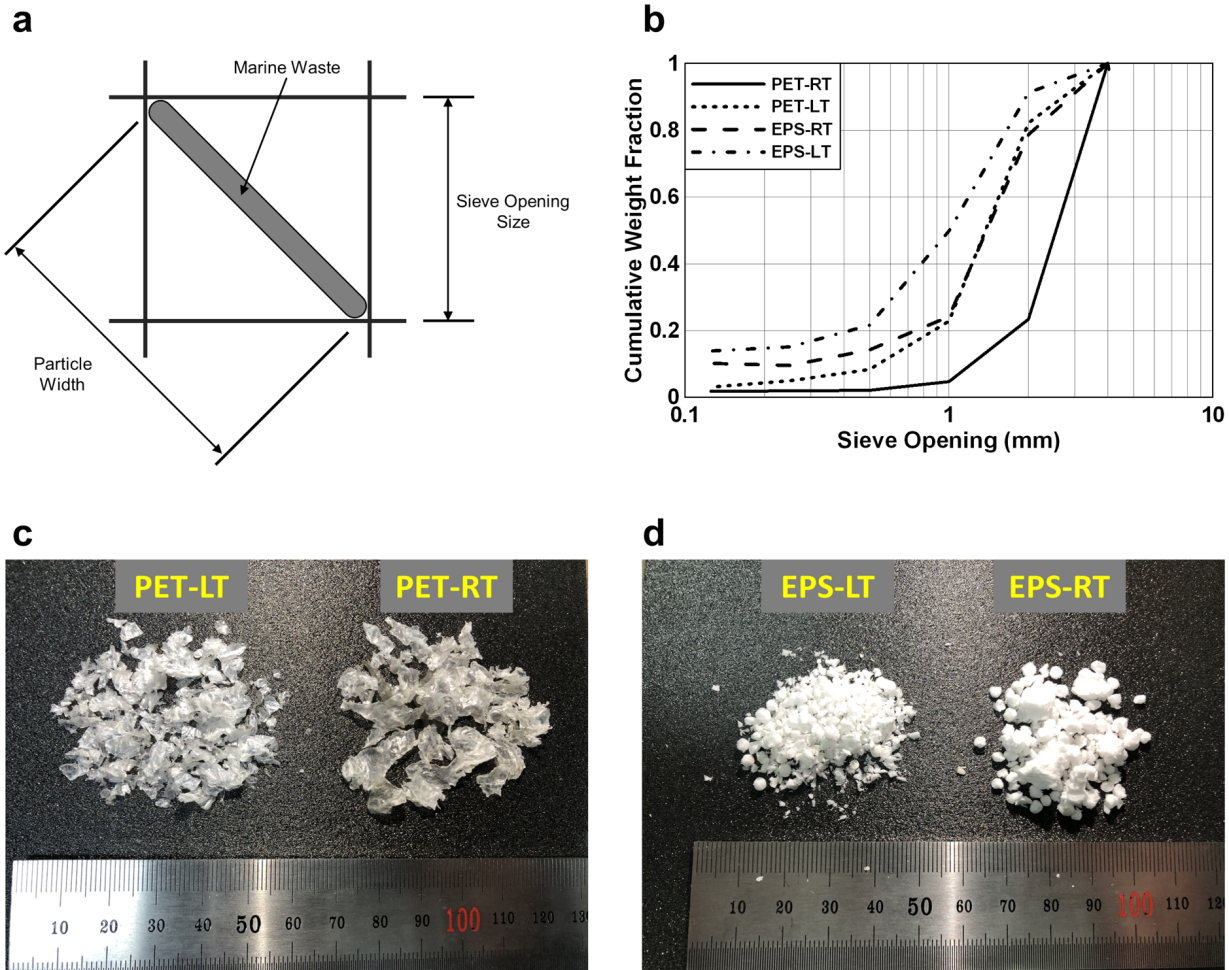
(**a**) Relationship between sieve opening size and particle size. Typically, MPD is not in a cube or spherical shape after grinding, so it is filtered up to particles equal to $\sqrt{2}$ times the sieve opening size. (**b**) Sieve test results. More than half of the PET-CT passed through the 1 mm sieve. (**c**,**d**) Photo of pulverized particles. In the case of PET-RT, the melted edges were observed, and the pulverized particles were coarser than PET-LT (**c**). In the case of EPS-RT, pulverization was performed in foam cell units (**d**).

A sieve test was conducted to analyze the pulverized particle size distribution. The sieve sizes were 0.25, 0.5, 1, 2, and 4 mm, respectively. For the uniformity of the sieve test, pulverizing was performed five times under the same conditions, and the intermediate data were used. The standard sieve had a squared mesh so that particles could pass through up to $\sqrt{2}$ times the mesh size for ground particles with irregular shapes as shown in Fig. 10a. Figure 10b shows the sieve test results.

The particle size may vary depending on the type of plastic, pulverizing time, and methods used. The target particle size of the LTP system was 5 mm and the results of the sieve tests showed that the typical particle size of MD after LTP was approximately 2–4 mm. In the case of PET particles pulverized at room temperature (PET-RT), about 76% of the particles failed to pass the largest sieve. In addition, melting and clumping around the edges were observed because of the high temperature of the pulverizing environment (Fig. 10c). PET particles pulverized at low temperature (PET-LT) around ~ 223 K showed no noticeable edge melting and clumping. In particular, 82% of the particles formed a particle size of less than 4 mm. Since EPS is produced by foaming polystyrene, it comprises a cell structure. The bond was broken between cells when it was ground even at room temperature, while the cell structure of EPS was crushed under low temperature (Fig. 10d). It is worth noting that rising temperatures in the pulverizing process cause plastic to melt. Due to the generation of a significant amount of endocrine-disrupting chemicals in this process, this should be resolved in the process of recycling^72^. In addition, existing crushing processes produce particles randomly distributed particle sizes, which degrades recycling quality. Uniform fine particles through LTP are eco-friendly and enable high-quality recycling.

## Summary

This study demonstrated a prototypical concept for an eco-friendly low-temperature MD pulverizing system that utilizes the cold energy from an LNG-powered cleaning ship. Typical cleaning ships used these days have a limited loading efficiency due to the low bulk density and larger volume of MD. As such, they mainly operate in coastal areas. However, the proposed concept can collect MD in the oceanic region because the MD loading capacity increases by more than 10 times through LTP and compression processes. Furthermore, the energy source for the LTP is mostly from excessive cold energy from LNG propulsion ships, which are essential for the upcoming low- CO_2_ emission requirement. It is also expected to dramatically reduce refrigerants used in LTP processes. By utilizing LCE at the ship’s designed speed (e.g., 11.5 knots in this study), it is expected that more than 2 tons of MD per hour can be frozen, and 200 kg of MD can be processed per hour even during collection at under 5 knots. In addition, the savings according to the ship's output were calculated by the cooling of MD using LN_2_. With an output of 20% MCR, more than 514 kg of MD can be processed per hour without consuming additional refrigerants. These results suggest that up to 253 kg of LN_2_ per hour can be saved during ship operation. To show the feasibility of the conceptual design, we estimated the amount of energy and fuel consumption needed for a proper cleaning capacity at various ship speeds. The outcomes are promising even though there is room for improvement. For example, the energy conversion efficiency and optimal system configurations in designing an LNG-powered cleaning ship can be improved with technological development. To evaluate the low-temperature process for adequate MD pulverization, residual particle size analysis was conducted. The results showed that LTP is advantageous for fine particle production and is eco-friendly by preventing melting. The pulverized MD particles can be utilized for upcycling and/or gasification through a dichlorination on board. This process could change the treatment process that relied on the existing collection to be environmentally friendly. As a result, the LTP system proposes an eco-friendly solution to the global marine pollution problem through eco-friendly ships (LNG-fueled propulsion ships). The proposed idea can resolve critical environmental issues in the ocean, but it can be generalized to ensure the utilization of any excess energy in modern industries.
